# Fist-Palm Test (FiPaT): a bedside motor tool to screen for global cognitive status

**DOI:** 10.1007/s10072-022-06129-1

**Published:** 2022-05-30

**Authors:** Sofia Cuoco, Roberto Erro, Immacolata Carotenuto, Marina Picillo, Maria Teresa Pellecchia, Paolo Barone

**Affiliations:** Department of Medicine, Surgery and Dentistry “Scuola Medica Salernitana”, Neuroscience Section, University of Salerno, Via Allende, 84131 Baronissi, SA Italy

**Keywords:** Cognitive screening, Mild cognitive impairment, Non-verbal test, Normal cognition

## Abstract

**Objective:**

The Fist-Palm Test (FiPaT) is a novel non-verbal task to be used at the patient’s bedside for a cognitive functions screening. The aims of this study are to analyze (I) the qualitative and quantitative performance features at FiPaT, (II) the psychometric characteristics of FiPaT, and (III) the correlation between FiPat and traditional cognitive assessments in subjects with normal cognition (NC), Mild Cognitive Impairment-single domain (MCI-sd), and Mild Cognitive Impairment-multiple domain (MCI-md).

**Methods:**

One hundred-thirteen subjects (53M/60F), with a mean age of 66.28 ± 7.22 years and 11.08 ± 4.93 years of education, were recruited and underwent a complete neuropsychological battery and FiPaT.

**Results:**

We found 68 subjects with NC, 31 with MCI-sd, and 14 with MCI-md and a high reliability of the FiPaT (alpha =0.762). The number of FiPaT errors correlated with age and all neuropsychological tests, except for the memory recall test. Subjects with MCI had greater FiPaT errors than subjects with NC. The FiPaT, used with the MOCA test, predicted the presence of MCI, with a variance of 44%.

**Conclusion:**

The FiPaT is an acceptable and reliable non-verbal test, able to screen for global cognitive status, attention, and executive functions, and to predict the MCI. Future studies will validate this initial findings as well as the discriminatory role of the FiPaT in detecting specific types of cognitive impairment.

**Supplementary Information:**

The online version contains supplementary material available at 10.1007/s10072-022-06129-1.

## Introduction

One of the principal aims of neuropsychology is to observe a behavior in a structured situation and to produce a score in order to transform empirical data into numerical data. Although the overlap between cognitive and motor functions has been abundantly recognized, there are very few standardized and validated motor tasks available, which can be used for screening global cognitive status or specific cognitive functions.

Alexander Luria proposed the most famous non-verbal test (i.e., imitation of the fist-cut-palm sequence), which could easily be performed by clinicians and is sensitive to executive function deficits associated with frontal lobe damage, but not to motor disorders [1, 2]. Luria’s main focus was largely qualitative: his test was highly flexible but was not standardized, which makes its reproduction between examiners difficult. Luria did not evaluate his sequence in neurodegenerative diseases but mainly in brain injuries [3, 4]. Furthermore, Luria’s sequence has never been standardized independently of the Frontal Assessment Battery (FAB), in which it was included with the aim of evaluating motor programming [1].

In this article, we describe the development and standardization of a novel non-verbal, programmed motor task, called the Fist-Palm Test (FiPaT), which is able to screen for cognitive deficits and might therefore be used to identify people who need in-depth neuropsychological assessments. Namely, the clinical objective of the FiPaT is to screen global cognitive status, with a focus on executive and attention functions, by means of a planned motor activity. Our goal is to validate a test and measure its properties, as well as its correlation with standard psychometric measurements. In fact, we aimed to link Luria’s more theoretical Russian approach with North American psychometric approaches to neuropsychology, connecting a part of clinical observation with a statistical analysis of the results [5]. Accordingly, we have analyzed the reliability of the test, its internal consistency in order to understand to what extent all its parts equally measure the variable, and its objectivity to ensure that the score is homogeneously assigned by two raters.

Through the FiPaT, we want to help clinicians with a short and easy-to-administer tool that is usable in different contexts [4]. To this aim, we here describe the qualitative and quantitative performance features of the FiPaT, its psychometric characteristics and its correlation with traditional cognitive assessment in healthy subjects or those with initial/mild cognitive impairment. Specifically, we included subjects with normal cognition (NC), Mild Cognitive Impairment-single domain (MCI-sd), and Mild Cognitive Impairment-multiple domain (MCI-md) as defined below, but the inclusion of the two latter served to compare the data against subjects with NC, in order to obtain normative data of the FiPaT. As a result, we obtained a lower representation of severe cases (i.e. MCI-md), as continued below.

## Methods

### Description of Fist-Palm Test (FiPaT)

The FiPaT was developed based on the clinical intuition of one of the authors (PB). Specifically, the FiPaT is a non-verbal test, composed of two sequences with 10 trials each. In each trial, the subjects are requested to successively place one on the other hand in two following postures: a fist resting vertically (i.e., Fist) and a palm resting horizontally (i.e., Palm). Therefore, for each of the two sequences, the subject must do the fist-palm combination (corresponding to what we call “a trial”) for ten consecutive times. In the first sequence, the subjects were requested to perform the task with their dominant hand, after having observed the examiner performing it for a complete sequence of 10 trials. The second sequence consists of the same task but performed with the other hand (i.e., with the non-dominant hand performing the task) and without the visual prompt provided by the examiner. Subjects were requested to refrain from reinforcing the motor task with any verbal cue such as counting 1–2-3… for each trial or verbalizing “fist-palm”. While the patient performs the task, the examiner must write down any errors on a notation sheet (Supplementary information S1). In the Supplementary information S2, we present the video of the delivery and correct execution of the FiPaT.

We considered the following qualitative errors:

1. Error in topography, in which the subjects made a mistake in the spatial orientation of the posture to perform (for instance, performed a palm trial vertically) but respected the sequence of the trials; (2) Perseverance, in which the subjects repeated an item more than once; (3) Attention, in which the subjects reversed the sequence of the test (i.e., performed a palm first) or made a single mistake and recovered; (4) Planning, when at the beginning of the sequences, the subjects were hesitant, producing wrong and/or inappropriate actions but could subsequently proceed with the correct task. We have arbitrarily assigned the following scores:

- 1) 0 = no error
- 2) 1 = error

The final score is given by the sum of all the errors present in Sequence_1 and Sequence_2, the minimum score being 0, which equates to a total correct performance, the maximum score being instead 8 and is equivalent to the presence of all types of error in both sequences.

### Sample

Consecutive subjects were recruited for the current study, between April 2018 and May 2019, from the neurological clinics of the Neuroscience Section, Department of Medicine, Surgery and Dentistry “Scuola Medica Salernitana,” University of Salerno, Italy, as well as among their healthy spouses.

The entire sample was composed of 113 subjects (53 M/60F) with 66.28 ± 6.52 years of age and 11.08 ± 4.93 years of education.

All subjects underwent a comprehensive neuropsychological battery and the FiPaT; subsequently, they were grouped on the basis of their cognitive status. We defined subjects with NC those who had at maximum only one abnormal test for any single domain at maximum [6, 7]. MCI was defined as the presence of at least two abnormal tests in a single (MCI-sd) or multiple domains (MCI-md) with preserved functional independence, whereas dementia diagnosis (which represented an exclusion criteria for this study) was defined by the presence of abnormal tests in one or more cognitive domains along with impairment of functioning/IADL, according to the Diagnostic and Statistical Manual for Mental Disorders, Fifth Edition (DSM-5) [8–10].

Therefore, the sample that we used in the subsequent analyses were composed of 68 subjects with NC, 31 MCI-sd, and 14 MCI-md. The means and standard deviations (SD) of the test scores reported by the subjects divided according to their cognitive status are visible in the Supplementary informationS3.

All participants gave written informed consent to participate, and the study was approved by the Local Ethics Committee.

### Cognitive assessment

Global cognitive abilities were screened with the Montreal Cognitive Assessment (MOCA) and its sub-scores [11]. Moreover, the subsequent neuropsychological tests were used: the immediate and delayed scores of the Rey Auditory Verbal Learning Test (15-RAWLT), the Copy and Recall of Rey Osterrieth Figure (ROCF), the Trail Making Test (TMT part A and part B), semantic fluency tests, the Frontal Assessment Battery (FAB), the Constructional Apraxia Test (CA) and Benton’s Judgment of Line Orientation (BJLO) [1, 12–14]. Functional autonomy was measured with the instrumental activities of daily life scale (IADL).

## Procedure and statistical analysis

### Performance at FiPaT and correlations with cognitive tests

In a preliminary analysis, we measured the number and types of errors on the FiPaT.

By χ^2^ test, we compared the percentage of correct performance between males and females and the percentage of qualitative errors in the entire sample and between male and females.

We calculated non-parametric Spearman’s correlations between number of errors and MOCA total score and sub-scores and between qualitative errors and MOCA total score and sub-scores.

We also calculated the Spearman’s correlation between qualitative errors and neuropsychological tests. Effect size of the correlation coefficients was defined with the following criteria: rho < 0.3 weak; rho = 0.3–0.5 moderate; rho > 0.5 strong.

## Relationship between the FiPaT and the global cognitive state and the presence and type of MCI

In order to analyze the qualitative error distribution, we stratified the entire sample as described below, conducted the Kruskal–Wallis, corrected with multiple comparisons, and the Mann–Whitney tests, were necessary. The sample was stratified as follows:

1. Altered MOCA score vs normal MOCA score [7 vs 106 subjects, respectively],
2. Normal cognition (NC) vs mild cognitive impairment (MCI) [68 vs 45 subjects, respectively],
3. Normal cognition (NC) vs mild cognitive impairment (MCI) with the involvement of a single domain (-sd) or of a multiple domain (-md) [68 vs 31 vs 14 subjects, respectively]
4. Normal cognition vs amnestic MCI-sd [68 vs 11 subjects, respectively]
5. Normal cognition vs executive MCI-sd [68 vs 11 subjects, respectively]

Since stratification based on variables, normal cognition against visuo-spatial MCI-sd (1/113) and normal cognition against attentional MCI-sd (0/113) were not possible due to a reduced presence of subjects in the experimental group, we instead used:

(6) MCI-md with attentional deficit vs MCI without attentional deficit [9 vs 35 subjects, respectively]
(7) MCI-md with visuo-spatial deficit vs MCI without visuo-spatial deficit [15 vs 28 subjects, respectively].

In order to investigate the degree of prediction of FiPaT, we used both logistic and linear regression models. Specifically, by binary logistic regressions, with stepwise backward method, we explored if the number of FiPaT errors, along with age, predicts the presence of altered MOCA score, MCI, memory, visual-spatial, attentional or executive alterations. To strengthen the FiPaT role as a screening assessment, we also performed a linear regression analysis, with stepwise methods, using age, FiPaT, and MOCA score as independent variables and presence/absence of MCI as dependent variable.

### Psychometric properties of FiPaT

Finally, we evaluated the internal consistency of the FiPaT, i.e., the extent to which the two sequences of the FiPaT reflect the same underlying construct, by calculating the Cronbach’s alpha coefficient. Internal consistency of the FiPaT was evaluated by Cronbach’s alpha; a value ≥ 0.70 was considered as acceptable. Quality of data obtained with the FiPaT was evaluated by computing percentage of missing or invalid items; a percentage of < 5% of missing values was considered as an index of acceptable data quality. Moreover, data quality was assessed using mean, median, skewness (criterion: − 1 to + 1) and extent of ceiling and floor effects. Floor and ceiling effects < 15% were defined as optimal. Scaling assumptions referring to the correct grouping of items and the appropriateness of their summed score were checked using corrected item-total correlation (standard ≥ 0.40).

The scores of two independent evaluators who were present during the administration of FiPaT conducted by one of them were compared and each evaluator was blind to the evaluations made by the other. The inter-rater reliability was determined by calculating the Kappa value and Pearson correlations. In order to examine the relative stability of FiPaT score, the intraclass correlation coefficient (ICC) between two raters was calculated.

All analyses were performed using SPSS version 20, (SPSS Inc., Chicago, IL, USA) with *p* value < 0.05 considered statistically significant.

## Results

### Performance at FiPaT and correlations with cognitive tests

**Fig. 1:**
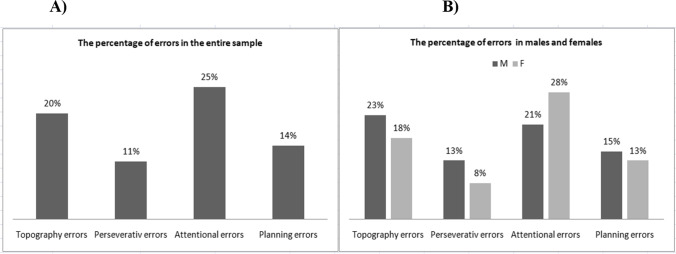
**A** The percentage of FiPaT errors in the entire sample. **B** The percentage of FiPaT errors in males and females. **Abbreviations:** F, females; FiPaT, Fist-Palm Test; M, males

The mean number (± SD) of errors was 0.929 ± 1.53. A total of 63% showed a perfect FiPaT performance, with no differences between males (36/53; 68%) and females (35/60; 58%) (*p* = 0.26). Regarding qualitative errors analysis, we found that 25% of the sample made attentional errors, 20% made topography errors, 14% made planning errors, and 11% made perseverative errors (Fig. 1A). There were no differences between male and female subjects in terms of qualitative errors (Fig. 1B, for all *p* > 0.05).

The number of errors correlated with age (rho = 0.305, *p* = 0.001) but did not correlate with education (rho =  − 0.047, *p* = 0.620). The errors number presented an inverse moderate correlation with the language score (rho =  − 0.301, *p* = 0.002), the attentional score (rho =  − 0.317, *p* = 0.001), the executive score (rho =  − 0.330, *p* = 0.001), the orientation score (rho =  − 0.336, *p* = 0.001), the visuo-spatial score (rho =  − 0.406, *p* < 0.001) and the MOCA total score (rho =  − 0.455, *p* < 0.001). The correlations between the number of errors and the scores of the neuropsychological tests are showed in Table 1.

**Table 1:** Correlations between number of FiPaT errors and neuropsychological tests

		**Number of errors**	
		**Rho**	***p***
**Executive domain**	FAB	− 0.362	** <0.001**
	Semantic fluency	− 0.354	** < 0.001**
	ROCF – Copy	− 0.274	** 0.005**
**Attentional domain**	TMT B	0.334	** 0.001**
	TMT A	0.332	** 0.001**
**Visuo- spatial domain**	BJLO	− 0.319	** 0.001**
	CA	− 0.291	** 0.002**
**Memory domain**	15-RAWLT- Immediate	− 0.262	** 0.006**
	ROCF- Recall	− 0.246	** 0.012**
	15-RAWLT- Recall	− 0.146	0.134

**Statistically significant results are in bold**

**Abbreviations:** *15-RAWLT*, Rey Auditory Verbal Learning Test; *BJLO*, Benton’s Judgment of Line Orientation; *CA*, Constructional Apraxia Test; *FAB*, Frontal Assessment Battery; *FiPaT*, Fist-Palm Test; *MOCA*, Montreal Cognitive Assessment; *ROCF*, Copy and Recall of Rey Osterrieth Figure; *TMT*, Trail Making Test

The correlations between the types of errors and the scores of the MOCA, sub-tests of MOCA, and whole neuropsychological battery are showed in Table 2. Specifically, correlations showed a significant association between topographical errors and all neuropsychological tests, but executive and attentional sub-tests of MOCA, BJLO, 15 RAWLT immediate and recall; between perseverative errors and all tests except language and attentional sub-tests of MOCA, BJLO, 15 RAWLT immediate and recall. There were significant correlations between attentional errors and all tests but ROCF-recall and except planning errors and language and orientation sub-tests of MOCA, TMT part A and B, BJLO, CA and 15 RAWLT immediate and recall (Table 2).

**Table 2:** Correlations between types of FiPaT errors and neuropsychological tests

			**Topography errors**	**Perseverative errors**	**Attentional errors**	**Planning errors**
**Global cognitive state and language and orientation sub-components**	MOCA total score	**Rho**	− 0.346	− 0.295	− 0.373	− 0.318
		**p**	** < 0.001**	** 0.002**	** < 0.001**	** 0.001**
	MOCA language sub-test	**Rho**	− 0.217	− 0.173	− 0.293	− 0.174
		**p**	** 0.029**	0.084	** 0.003**	0.081
	MOCA orientation sub-test	**Rho**	− 0.341	− 0.237	− 0.324	− 0.095
		**p**	** < 0.001**	** 0.017**	** 0.001**	0.346
**Executive domain**	MOCA executive sub-test	**Rho**	− 0.174	− 0.264	− 0.344	− 0.199
		**p**	0.081	** 0.008**	** <0 .001**	** 0.046**
	FAB	**Rho**	− 0.263	− 0.217	− 0.281	− 0.297
		**p**	** 0.006**	** 0.024**	** 0.015**	** 0.002**
	Semantic fluency	**Rho**	− 0.199	− 0.228	− 0.358	− 0.199
		**p**	** 0.046**	** 0.022**	** < 0.001**	** 0.046**
	ROCF- Copy	**Rho**	− 0.225	− 0.278	− 0.238	− 0.200
		**p**	** 0.021**	** 0.004**	** 0.015**	** 0.041**
**Attention domain**	MOCA attention sub-test	**Rho**	− 0.179	− 0.132	− 0.308	− 0.223
		**p**	0.073	0.189	** 0.002**	** 0.025**
	TMT A	**Rho**	0.232	0.219	0.330	0.092
		**p**	** 0.019**	** 0.028**	** 0.001**	0.362
	TMT B	**Rho**	0.237	0.323	0.343	0.178
		**p**	** 0.019**	** 0.001**	** 0.001**	0.082
**Visuo-spatial domain**	MOCA visuo- spatial sub-test	**Rho**	− 0.246	0.285	− 0.380	− 0.271
		**p**	** 0.013**	** 0.004**	** < 0.001**	** 0.006**
	BJLO	**Rho**	− 0.186	− 0.178	− 0.284	− 0.135
		**p**	0.064	0.076	** 0.004**	0.179
	CA	**Rho**	− 0.250	− 0.239	− 0.291	− 0.180
		**p**	** 0.010**	** 0.014**	** 0.002**	0.065
**Memory domain**	15-RAWLT- Immediate	**Rho**	− 0.174	− 0.128	− 0.293	− 0.122
		**p**	0.074	0.188	** 0.002**	0.209
	ROCF- Recall	**Rho**	− 0.208	− 0.246	− 0.162	− 0.333
		**p**	** 0.035**	** 0.012**	0.101	** 0.001**
	15-RAWLT- Recall	**Rho**	− 0.128	− 0.033	− 0.237	0.020
		**p**	0.189	0.736	** 0.014**	0.838

**Statistically significant results are in bold**

**Abbreviations:** *15-RAWLT*, Rey Auditory Verbal Learning Test; *BJLO*, Benton’s Judgment of Line Orientation; *CA*, Constructional Apraxia Test; *FAB*, Frontal Assessment Battery; *FiPaT*, Fist-Palm Test; *MOCA*, Montreal Cognitive Assessment; *ROCF*, Copy and Recall of Rey Osterrieth Figure; *TMT*, Trail Making Test

## Relationship between the FiPaT, the global cognitive state, and the presence and type of MCI

Subjects with MOCA score below the cut-off showed a greater number of total errors (*p* < 0.001), topography (*p* < 0.001), perseverative (*p* = 0.003), attentional (*p* = 0.004), and planning (*p* < 0.001) errors than subjects with normal MOCA score. Subjects with MCI showed a greater number of total errors (*p* = 0.001), topography (*p* = 0.002), attentional (*p* = 0.03), and planning (*p* < 0.001) errors, but not perseverative errors (*p* = 0.14) than NC subjects.

There were significant differences between NC, MCI-sd, and MCI-md in total errors (*p* < 0.001), topography (*p* < 0.001), attentional (*p* = 0.01), and planning (*p* < 0.001) errors and not in perseverative errors (*p* = 0.31). After correction for multiple comparisons (*p* = 0.013), MCI-md had greater total errors (*p* < 0.001), topography (*p* < 0.001), attentional (*p* = 0.003), and planning (*p* < 0.001) errors than NC. On the other hand, MCI-sd showed greater planning errors than NC (*p* = 0.001) and MCI-md made more topography errors than MCI-sd (*p* < 0.01). Subjects with amnesic MCI-sd showed only planning score higher than NC (*p* = 0.03). Subjects with executive MCI-sd had higher perseverative (*p* = 0.02) and planning errors (*p* < 0.001) than NC. There were no significant differences between MCI-md with visuo-spatial and MCI-md without visuo-spatial deficit (*p* > 0.05). MCI-md with attentional deficit subjects showed greater total errors (*p* = 0.02), topography (*p* < 0.001) and attentional errors (*p* = 0.04) than subjects with MCI-md without attentional domain alterations (Supplementary information S4).

In order to assess the predictive role of FiPaT, the logistic regression model showed that the FiPaT predicted alterations of MOCA, presence of MCI, MCI with executive isolated alterations, and MCI with attentional involvement; whereas age was never a co-predictor (*p* < 0.05). The results of number of FiPaT errors role are showed in Table 3. Moreover, FiPaT (β =  − 0.25, *p* = 0.01) and MOCA score (β = 0.26, *p* = 0.01), but not age (β = 0.11, *p* = 0.24), were significant predictors of MCI, explaining about 44% of the variance (*R*^2^ = 0.44).

**Table 3:** Binary logistic regression data demonstrating the predictive role of FiPaT on cognitive status

Dipendent variables	*R*^2^	B	P
**MOCA score > 15.5** vs **MOCA score ≤ 15.5**	0.482	− 1.030	** <0.001**
**MCI vs NC**	0.194	− 0.623	** < 0.001**
**MCI-sd** with **amnesic deficit** vs **NC**	0.054	− 0.487	0.108
**MCI-md** with **Attentional deficit** vs **MCI** without **Attentional deficit**	0.218	− 0.531	** 0.015**
**MCI-sd** with **Executive deficit** vs **NC**	0.155	− 0.677	** 0.008**
**MCI-md** with **Visuo-spatial deficit** vs **MCI** without **Visuo-spatial deficit**	0.126	− 0.326	* 0.049*

**Statistically significant results are in bold**

**Abbreviations:** *FiPaT*, Fist-Palm Test; *MCI*, Mild Cognitive Impairment; *md*, multiple domain; *MOCA*, Montreal Cognitive Assessment; *NC*, normal cognition; *sd*, single domain

### Psychometric properties of FiPaT

With regard to the acceptability of the test, we found that the 100% of data were totally computable. Lowest possible score was 0 (63%), highest possible score was 6 (3%). Skewness of FiPaT was out of the standard limits (score = 1.75). The Cronbach’s alpha, used to measure the reliability of FiPaT, was 0.762 indicating a high level of internal consistency. Specifically, the items topography_1, topography_2, attention_1, attention_2, and planning_2 had strong Spearman’s correlations with the FiPaT total score (rho > 0.50, *p* < 0.01); all other items showed a moderate correlation with the FiPaT total score (rho > 0.40, *p* < 0.01). Regarding the relative stability, we found that the agreement between the two blinded raters was 75%; the Kappa value, used to confirm inter-rater reliability, was 0.619 (*p* < 0.001). The CCI between scores administered by two raters was 0.793 (0.669–0.874, confidence interval for 95%; *p* < 0.01).

## Discussion

We have described the development and the psychometric properties of a novel non-verbal, programmed motor task, able to screen for global cognitive status, attention and executive functions, and to be used to identify subjects that may need a more comprehensive neuropsychological assessment.

The FiPaT is to be intended as a neuropsychological screening test that, differently from traditional pen-and-paper–based neuropsychological tests, can be easily used bedside. Similar to Luria’s test, the FiPaT is a non-verbal programmed motor task, but offers the advantage of being a pure quantitative test, further providing structured details on both qualitative and quantitative evaluations. Moreover, the FiPaT has been developed as an independent test, while Luria’s Test is mostly considered in relation to FAB [1].

The internal consistency of the FiPaT is high and acceptable (alpha = 0.762), the temporal stability of scores is good (ICC = 0.793) and the agreement between the two blinded raters (75%) and Kappa value (0.619) are high.

We found that 63% of the whole sample have a good performance at FiPaT which almost equates to the 60.2% of NC identified through formal neuropsychometry. The FiPaT is not affected by the sex variable, probably because the cognitive component weighs more than the motor one. Accordingly, previous literature does not report sex differences for executive and attentional functions [15, 16]. Furthermore, the number of errors correlates with age and not with education and this is in line with the natural course of brain skills [17]. Regarding qualitative analysis, we found that attentional errors are the most frequent errors, especially in females, as reported for other attentional neuropsychological tests [18].

Analyzing the correlations between the number of errors and neuropsychological tests, the highest correlation was found with the MOCA total score, and this strengthens the FiPaT function as a screening tool to investigate global cognitive status. The MOCA test, in fact, simultaneously evaluates different cognitive functions [19]. Moreover, we found that the FiPaT correlated with many single neuropsychological tests and this reinforces its role as a screening task of the global cognitive status.

The convergent validity of the FiPaT is exemplified by its high correlation with the executive and attention domains, which are also the predominant domains represented in the MOCA [19]. Specifically, visual-motor coordination, mental flexibility, processing speed, inhibitory control, divided attention are the specific executive and attention functions that most correlate with the FiPaT. On the other hand, the absence of correlation between memory and FiPaT confirms the divergent validity. We also observe a low correlation between FiPaT and planning test, due to the involvement of different subcomponents of planning skills [20]. Furthermore, attention errors are related to all functions measured by tests, and planning errors are the only ones related to a single domain, such as the executive domain. It should be noted, however, that there are no tests measuring specifically one single cognitive function. Therefore, the choice we made to select some particular neuropsychometry tests to measure the different types of cognitive functions might theoretically affect subjects classification and the results [21]. However, we note that this choice was based on current literature [21–28], and future studies addressing patients with specific clinical cognitive syndromes will better clarify the relative contribution of different cognitive functions on the FiPaT performances.

Finally, FiPaT appears to be a predictor of cognitive impairment. In fact, subjects with worse cognitive status have significantly higher FiPaT scores than NC and the FiPaT, used with the MOCA test, predicted the presence of MCI in our sample, with a variance of 44%. Given its preferential ability to detect executive/attentional deficits, it might be argued that the FiPaT might be able to further discriminate between different types of MCI (i.e., amnestic vs non-amnestic), which in turn are construed to be predictive of different types of dementia [29]. Future studies will validate this initial findings as well as the discriminatory role of the FiPAT in detecting specific types of cognitive impairment.

## Supplementary Information

Below is the link to the electronic supplementary material.

Supplementary file1 (DOCX 16 kb)

Supplementary file2 (MP4 109212 kb)

Supplementary file3 (DOCX 15 kb)

Supplementary file4 (DOCX 67 kb)

## Data Availability

The datasets generated during and/or analysed during the current study are available from the corresponding author on reasonable request.
