# Supplementary material for: Fist-Palm Test (FiPaT): a bedside motor tool to screen for global cognitive status
Source: Neurol Sci. 2022 May 30;43(9):5251–8. doi: 10.1007/s10072-022-06129-1 (PMC9385767; doi:10.1007/s10072-022-06129-1)
Supplement: Supplementary file 1 — Supplementary file1 (DOCX 16 kb) [file 10072_2022_6129_MOESM1_ESM.docx]

**Supplementary Material-S1**:

**Fist-Palm Test (FiPaT)**

“Watch carefully what I do, then you do the same”. After the first sequence, the examiner says “Now change hands”.

|  | **Trial_1** | **Trial_2** | **Trial_3** | **Trial_4** | **Trial_5** | **Trial_6** | **Trial_7** | **Trial_8** | **Trial_9** | **Trial_10** | Errors |
| --- | --- | --- | --- | --- | --- | --- | --- | --- | --- | --- | --- |
| **Sequence_1** |  |  |  |  |  |  |  |  |  |  | - Topography (0-1) - Perseverance (0-1) - Attention (0-1) - Planning (0-1)   Total S_1: _____/4 |
| Note S_1 |  | | | | | | | | | |  |
|  | **Trial_1** | **Trial_2** | **Trial_3** | **Trial_4** | **Trial_5** | **Trial_6** | **Trial_7** | **Trial_8** | **Trial_9** | **Trial_10** |  |
| **Sequence_2** |  |  |  |  |  |  |  |  |  |  | - Topography (0-1) - Perseverance (0-1) - Attention (0-1) - Planning (0-1)   Total S_2: _____/4 |
| Note S_2 |  | | | | | | | | | |  |
| **Total score: _____/8** | | | | | | | | | | | |

*Definitions:*

1) Error in topography, in which the subjects made a mistake in the spatial orientation of the posture to perform (for instance, performed a palm trial vertically) but respected the sequence of the trials; 2) Perseverance, in which subjects repeated an item more than once; 3) Attention, in which subjects reversed the sequence of the test (i.e., performed a palm first) or made a single mistake and recovered; 4) Planning, when at the beginning of the sequences subjects were hesitant, producing wrong and/or inappropriate actions but could subsequently proceed with the correct task.

Scores:

1) 0 = no error

2) 1 = error

(Cuoco et al., 2022)
