# Supplementary material for: Fist-Palm Test (FiPaT): a bedside motor tool to screen for global cognitive status
Source: Neurol Sci. 2022 May 30;43(9):5251–8. doi: 10.1007/s10072-022-06129-1 (PMC9385767; doi:10.1007/s10072-022-06129-1)
Supplement: Supplementary file 3 — Supplementary file3 (DOCX 15 kb) [file 10072_2022_6129_MOESM3_ESM.docx]

**Supplementary Material-S3:** The means and standard deviations of the test scores reported by the subjects divided according to their cognitive status

|  |  | **NC**  (N= 68)  Mean±SD | **MCI-sd**  (N= 31)  Mean±SD | **MCI-md**  (N= 14)  Mean±SD |
| --- | --- | --- | --- | --- |
| **Global cognitive status** | MOCA | 23.18±4.08 | 21.30±3.86 | 15.23±4.14 |
| **Executive domain** | FAB | 14.95±2.55 | 13.21±2.71 | 9.84±2.33 |
|  | Semantic fluency | 37.48±11.50 | 31.24±12.33 | 19.56±7.86 |
|  | ROCF – Copy | 29.77±7.57 | 26.04±6.54 | 14.88±7.16 |
| **Attentional domain** | TMT A | 49.80±22.37 | 65.53±27.34 | 111.00±62.75 |
|  | TMT B | 127.20±70.48 | 192.08±111.44 | 275.14±147.78 |
| **Visuo- spatial domain** | BJLO | 20.96±5.13 | 17.08±6.40 | 12.10±5.30 |
|  | CA | 11.76±1.81 | 10.53±1.90 | 7.61±2.56 |
| **Memory domain** | 15-RAWLT- Immediate | 37.43±9.78 | 30.73±8.25 | 21.30±6.77 |
|  | 15-RAWLT- Recall | 7.73±3.21 | 5.85±3.09 | 3.57±2.11 |
|  | ROCF- Recall | 15.62±7.65 | 10.80±7.10 | 5.04±3.04 |

**Abbreviations:** 15-RAWLT, Rey Auditory Verbal Learning Test; BJLO Benton’s Judgment of Line Orientation; CA, Constructional Apraxia Test; FAB, Frontal Assessment Battery; MCI, Mild Cognitive Impairment; md, multiple domain; MOCA, Montreal Cognitive Assessment; N, number; NC, normal cognition; ROCF, Rey Osterrieth Figure; sd, single domain; SD, standard deviation; TMT, Trail Making Test.
