# Supplementary material for: Fist-Palm Test (FiPaT): a bedside motor tool to screen for global cognitive status
Source: Neurol Sci. 2022 May 30;43(9):5251–8. doi: 10.1007/s10072-022-06129-1 (PMC9385767; doi:10.1007/s10072-022-06129-1)
Supplement: Supplementary file 4 — Supplementary file4 (DOCX 67 kb) [file 10072_2022_6129_MOESM4_ESM.docx]

**Supplementary Material-S4:** Comparison of the number errors of FiPaT, between groups.


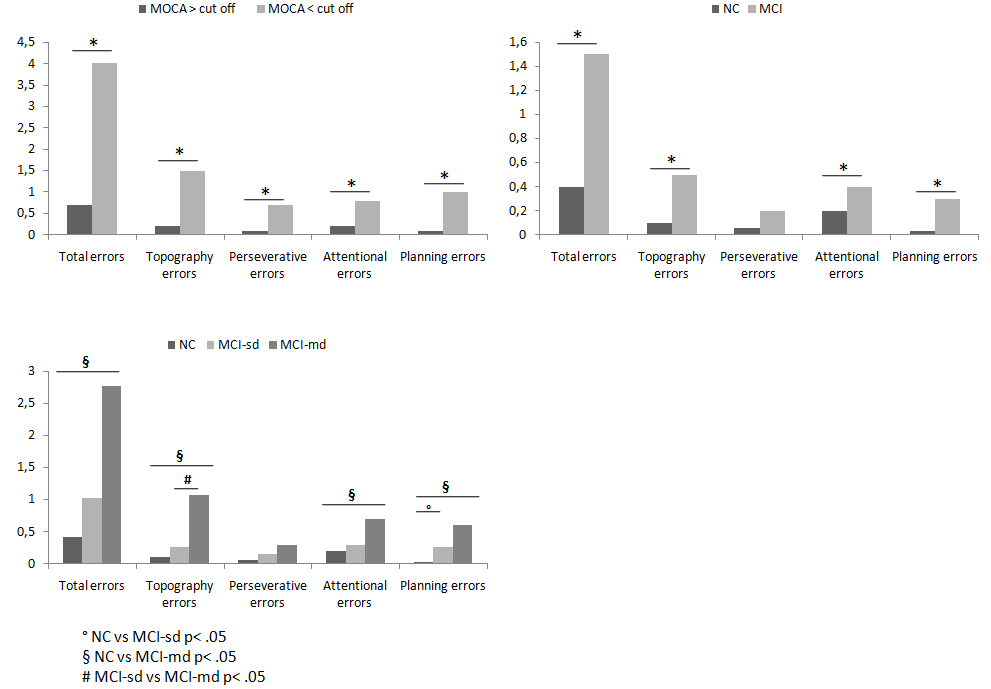


*** Statistically significant results * Statistically significant results**


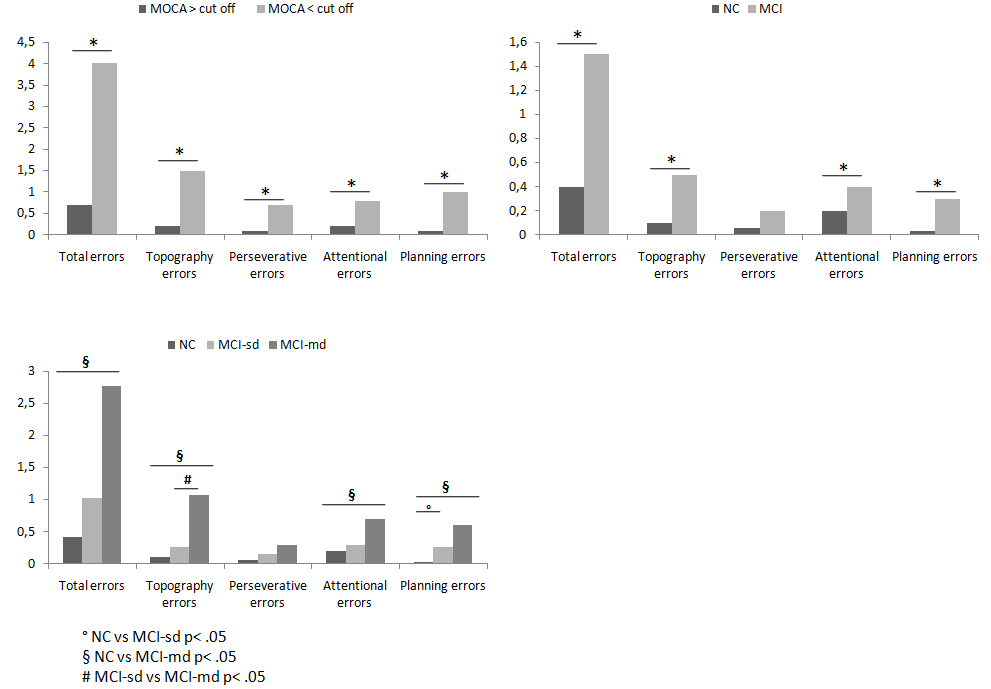


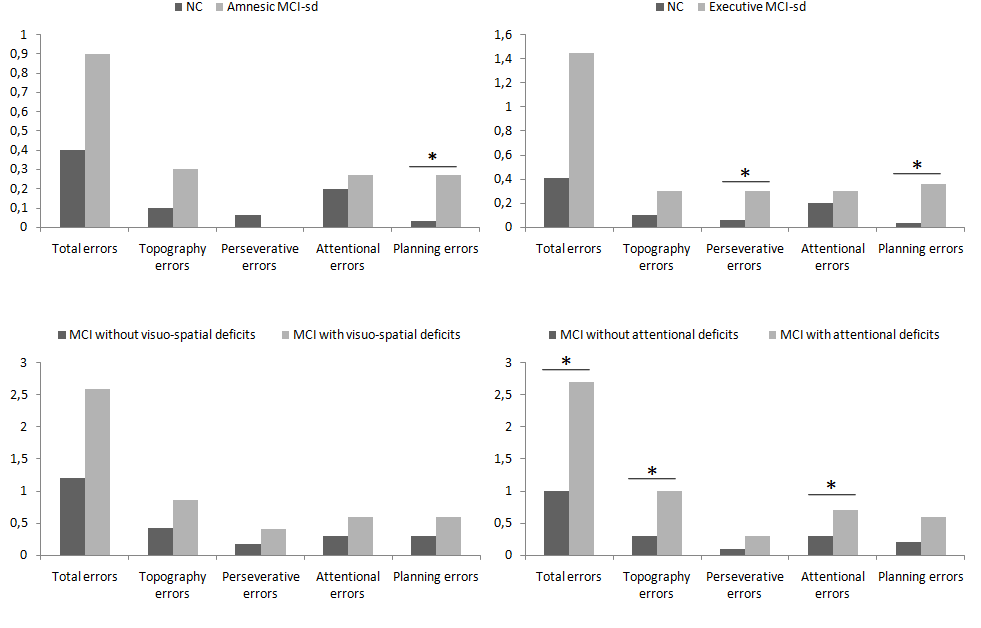


*** Statistically significant results * Statistically significant results**


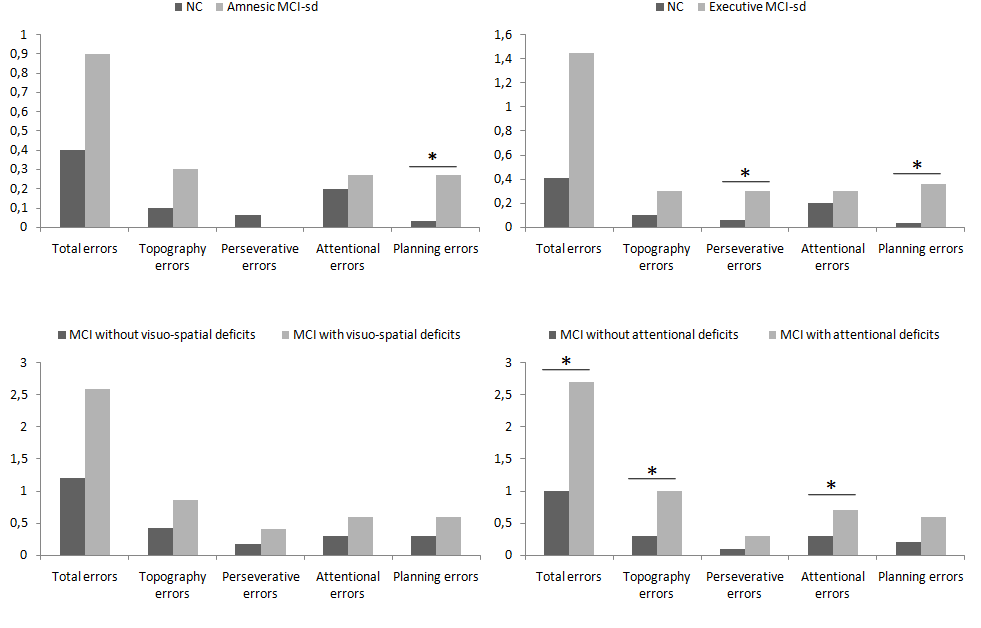


*** Statistically significant results * Statistically significant results**

**Abbreviations:** FiPaT, Fist-Palm test; MCI, Mild Cognitive Impairment; md, multiple domain; MOCA, Montreal Cognitive Assessment; NC, normal cognition; sd, single domain.
